# Visualizing polymeric components that define distinct root barriers across plant lineages

**DOI:** 10.1242/dev.199820

**Published:** 2021-12-08

**Authors:** Moritz Sexauer, Defeng Shen, Maria Schön, Tonni Grube Andersen, Katharina Markmann

**Affiliations:** 1 Department of Plant Physiology, Zentrum für Molekularbiologie der Pflanzen, Tübingen University, 72076 Tübingen, Germany; 2 Department of Plant Microbe Interactions, Max Planck Institute for Plant Breeding Research, 50829 Cologne, Germany

**Keywords:** Suberin, Lignin, Endodermis, Periderm, ClearSee, Fluorol Yellow, Basic Fuchsin, Symbiosis

## Abstract

Hydrophobic cell wall depositions in roots play a key role in plant development and interaction with the soil environment, as they generate barriers that regulate bidirectional nutrient flux. Techniques to label the respective polymers are emerging, but are efficient only in thin roots or sections. Moreover, simultaneous imaging of the barrier constituents lignin and suberin remains problematic owing to their similar chemical compositions. Here, we describe a staining method compatible with single- and multiphoton confocal microscopy that allows for concurrent visualization of primary cell walls and distinct secondary depositions in one workflow. This protocol permits efficient separation of suberin- and lignin-specific signals with high resolution, enabling precise dissection of barrier constituents. Our approach is compatible with imaging of fluorescent proteins, and can thus complement genetic markers or aid the dissection of barriers in biotic root interactions. We further demonstrate applicability in deep root tissues of plant models and crops across phylogenetic lineages. Our optimized toolset will significantly advance our understanding of root barrier dynamics and function, and of their role in plant interactions with the rhizospheric environment.

## Abstract (precis)

**Summary:** A toolset enabling combined imaging of suberin and lignin, two cell wall polymers controlling molecular exchange between plant root and rhizosphere, is described and demonstrated to be robust across plant lineages.

## INTRODUCTION

Roots are complex, dynamic organs that facilitate the extraction of solutes from their surroundings and mediate plant interactions with the biotic soil environment. In contrast to above-ground plant organs of most vascular plants, roots feature a central vascular cylinder known as the stele, which contains conductive xylem and phloem tissue responsible for bidirectional long-distance transport of water, minerals and assimilated solutes. The stele is surrounded and protected by concentric cell files including a highly specialized cell layer known as the endodermis, as well as outer cortical cell layers that vary in number between plant lineages. The endodermis directly surrounds the stele and serves as a dynamic filter, providing control over radial transport of solutes to and from the vascular tissues (Barberon and Geldner, 2014; Geldner, 2013). This sophisticated function is facilitated by the establishment of diverse polymeric secondary cell wall depositions. At the periphery of the cortex, often right underneath the outermost root epidermis, certain plant lineages feature a cell layer termed the exodermis, which is reminiscent of the endodermis in structure and function (Enstone et al., 2002). Arguably, the best-known endodermal barrier is the Casparian strip, which consists of defined, ring-shaped cell wall depositions of lignin synthesized autonomously in the endodermis (Andersen et al., 2021; Naseer et al., 2012). The Casparian strip blocks apoplastic diffusion to and from the rhizosphere in a manner that is remarkably similar to tight junctions in animals (Doblas et al., 2017), providing control of radial flow of water and solutes. As the endodermis matures in older root parts, hydrophobic suberin is deposited on the entire surface of most endodermal cell walls, blocking transcellular transport across the endodermal plasma membrane. In contrast to the Casparian strip, which consists of phenylpropanoid-derived lignin monomers (Naseer et al., 2012), suberin contains both aromatic and aliphatic constituents (Schreiber, 2010).

The deposition of lignin- or suberin-containing barriers in roots is not limited to the endodermis, they can also be found in the exodermis or in the periderm. The periderm is a frontier tissue developed during secondary growth of most eudicots and gymnosperms (Ragni and Greb, 2018). After it replaces the epidermis as the outermost tissue, the periderm restricts water and gas exchange (Lendzian, 2006), and grants resistance to pathogens (Lulai and Freeman, 2001). The patterning of lignin- or suberin-containing barriers, in terms of design and extent, is dynamic and varies between tissues, as well as between lineages and species. Although much remains to be understood about the genetic control and dynamics of barriers in roots, emerging evidence suggests that they play a key role in defining molecular communication with the underground environment, and also in shaping associated microbial communities (Salas-González et al., 2021).

The spatiotemporal differences in suberin and lignin deposits suggest that their roles in plant development and adaptation to environmental factors differ. To investigate this at a functional level, it is thus fundamental to visualize the individual barrier components differentially. Currently established methods rely on either autofluorescence, Raman signal or histochemical dyes (Rydahl et al., 2018; Wallace and Anderson, 2012) that can specifically highlight lignin or suberin, such as Basic Fuchsin (BF) and Fluorol Yellow (FY), respectively. One key limitation is that neither of these tools allows simultaneous visualization of lignin and suberin because of overlap in their emission spectra (DeVree et al., 2021) or incompatibility of the respective histochemical procedures (Ursache et al., 2018). Approaches based on genetically encoded fluorescent transcriptional reporters (Andersen et al., 2018; Barberon et al., 2016) have partially overcome this, but are limited to the underlying machinery and to genetically tractable models such as *Arabidopsis thaliana*. Moreover, although *A. thaliana* indeed is an outstanding model for image analysis and root developmental biology research, it lacks endosymbiotic associations, such as fungal arbuscular mycorrhiza formation or nitrogen-fixing nodulation with bacteria. As the vast majority of land plants form either fungal or bacterial root symbioses, such interactions are of extensive ecological and economical significance, and the current limitations therefore hinder the detailed study of the role of barriers in a biotic context.

Here, we present an improved histochemical staining technique that can distinguish lignin from suberin with subcellular resolution and a high degree of specificity. Our method is compatible with fluorescent markers and widely applicable to roots of varying thickness and complexity. We use this to highlight differences in barrier-associated lignin and suberin depositions in roots across diverse phylogenetic lineages, including both model and crop species. Compatibility of the toolset with imaging of fluorescent dyes and microbial markers make it a prime tool for hydrophobic barrier analysis also in root symbiotic contexts.

## RESULTS AND DISCUSSION

**Fig. 1.**
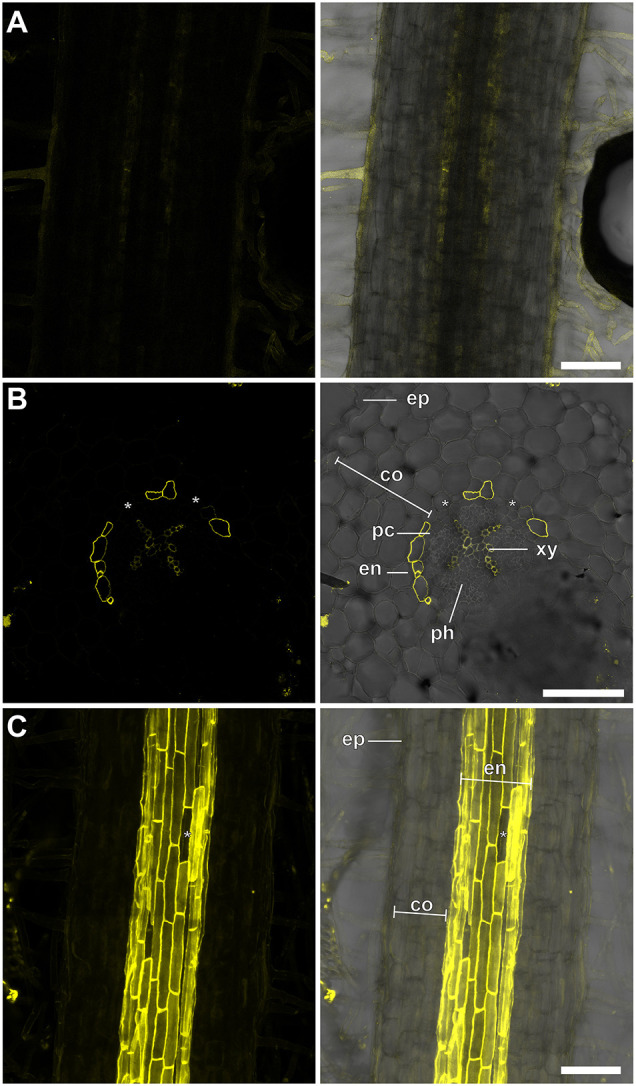
**Staining of endodermal suberization in *L.*** ***japonicus*****.** (A,B) FY-stained *L. japonicus* whole-mount roots (A) and semi-thin sections (B) using a lactic acid-based protocol. (C) *L. japonicus* roots stained using the optimized ClearSee-based method. Asterisks indicate passage cells. Left panels: FY channel. Right panels: merged FY and transmission light channels. Plants grew for 10 days. co, parenchymatic cortex; en, endodermis ep, epidermis; pc, pericycle; ph, phloem; xy, xylem. Scale bars: 100 µm.

Aiming to visualize specifically endodermal suberin depositions in roots of different model and crop plants for comparative analysis, we initially used a well-established lactic acid-based protocol for FY staining of suberin in *Arabidopsis thaliana* roots and semi-thin cuts (Lux et al., 2005). When applied to *Lotus japonicus* roots, which have a different internal structure and more cortical cell layers than *A. thaliana* roots, this protocol did give rise to suberin-associated signals (Fig. 1A,B), but these were weak and difficult to image owing low signal intensity in whole-mount roots (Fig. 1A). To improve staining in deeper root tissues, we combined the lactic acid-based FY staining directly with a previously established ClearSee-based protocol (Kurihara et al., 2015), which has been successfully used together with other histochemical dyes (Ursache et al., 2018). However, this approach resulted in a precipitation of FY and almost complete loss of suberin-associated signals. To solve this, we tested alternative solvents for FY, and found that the use of 96% ethanol rendered the staining solution compatible with ClearSee. A combined treatment with ethanol-dissolved FY and ClearSee yielded greatly enhanced signals from suberized endodermal cells in *L. japonicus* roots (Fig. 1C) compared with the initially tested protocol (Lux et al., 2005) (Fig. 1A).

**Fig. 2.**
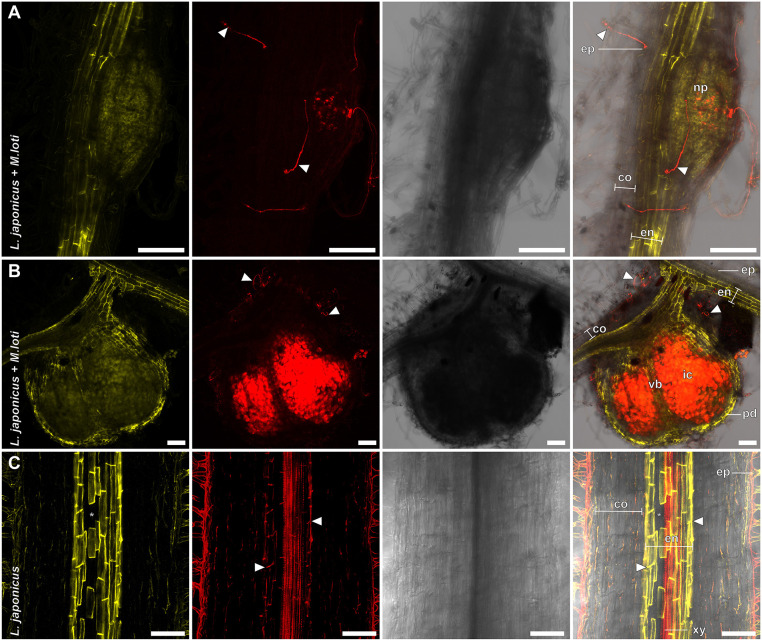
**FY-based suberin staining is compatible with BF staining of lignin and fluorescent protein imaging.** (A-C) *L. japonicus* root showing nodule primordia prior to formation of a suberized periderm (10 days post-inoculation), (B) mature nodule (21 days post-inoculation) and (C) uninfected root 10 days post-germination stained with FY (A,B) or double-stained with FY and BF (C) using an optimized ClearSee-based method. Panels (left to right) show FY; DsRED (A,B) or BF (C); transmission light; and merged channel. (A,B) *L. japonicus* infected with *M. loti* expressing DsRED at 10 and 21 days post-inoculation, respectively. (C) Root co-stained with BF. White arrowheads indicate infection threads (A,B) or Casparian strip (C). Asterisks indicate passage cells. co, parenchymatic cortex; en, endodermis; ep, epidermis; ic, infected nodule cortex; np, nodule primordia; pd, periderm; xy, xylem; vb, vascular bundle. Scale bars: 100 µm.

In contrast to the established lactic acid-based protocol, this procedure can be performed at room temperature, suggesting that it may be compatible with imaging of fluorescent proteins. To test this, we employed a *DsRED*-expressing strain of the rhizobacterium *Mesorhizobium loti*, which symbiotically infects *L. japonicus* roots. This setting further enabled us to evaluate whether these bacteria remained traceable in FY-stained roots, and to test the applicability of the protocol for analyzing barriers in a root endosymbiotic context (Fig. 2A,B). Intriguingly, suberized endodermal cells and *DsRED*-labelled epidermal and cortical infection threads could be reliably visualized in the same samples (Fig. 2A). Furthermore, a suberized periderm-like layer in colonized nodules was apparent. In line with earlier observations in *Vicia faba* nodules (Hartmann et al., 2002), these suberized cells appeared to connect to the endodermis of the root tissue and of nodule vascular bundles (Fig. 2B). This hints towards an important function of cell wall barriers in this plant-bacterial interaction, and paves the way for in-depth visual analysis of suberin depositions in the context of nodulation symbiosis.

**Fig. 3.**
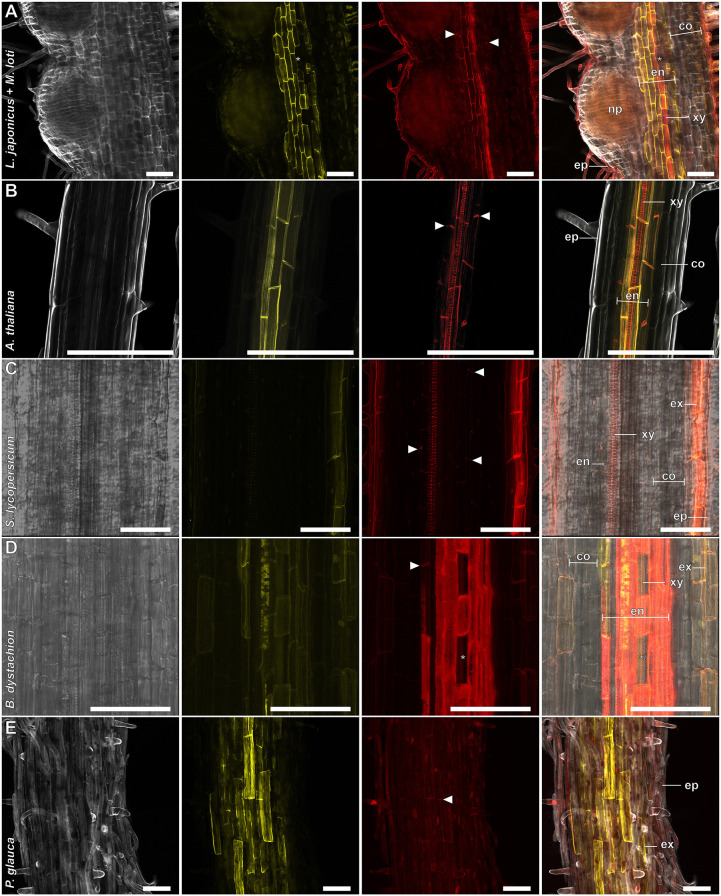
**Visualization of root barriers in a broad range of seed plants.** (A-E) Whole-root mounts of *L. japonicus* infected with *M. loti* showing pre-peridermal nodule primordia (10 days post-inoculation) (A), *A. thaliana* (B), *S. lycopersicum* (C), *B. distachyon* (D) and *P. glauca* (E). Roots were stained with BF, FY and CW (A,B,E only). Panels (left to right) show CW (A,B,E) or transmission light (C,D); FY; BF; and merged channels. White arrowheads indicate Casparian strip. Asterisks indicate passage cells. Plants grew for 14 (A,E) or 10 (B,C,D) days. co, parenchymatic cortex; en, endodermis; ep, epidermis; ex, exodermis; np, nodule primordia; xy, xylem. Scale bars: 100 µm.

**Fig. 4.**
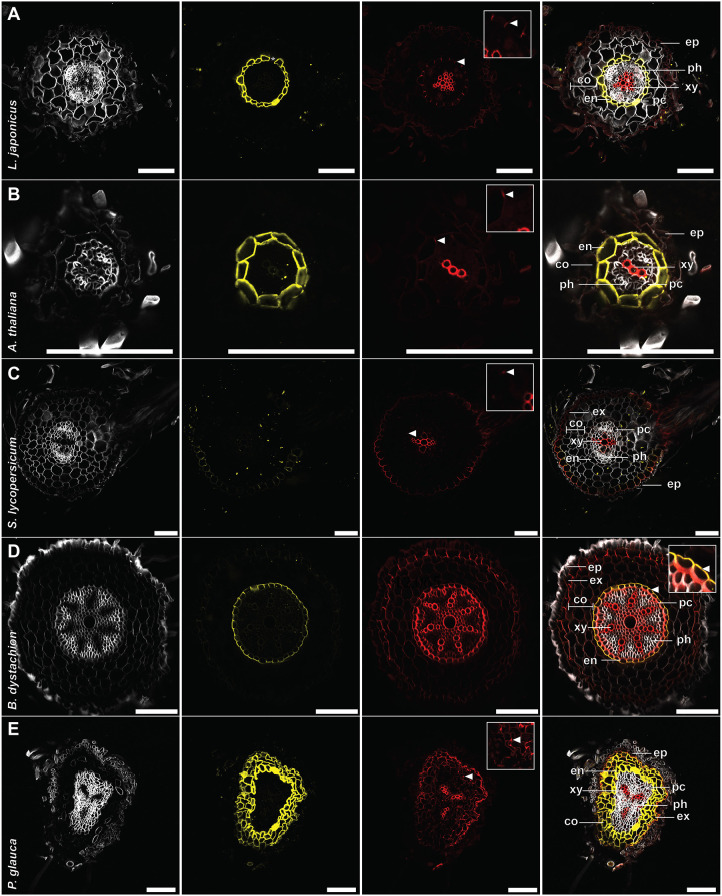
**Triple staining of cell wall components in semi-thin cross-sections of seed plant roots.** (A-E) BF, FY and CW triple-stained cross-sections of *L. japonicus* (A), *A. thaliana* (B), *S. lycopersicum* (C), *B. distachyon* (D) and *P. glauca* roots (E). Panels (left to right) show CW; FY; BF; and merged channels. White arrowheads indicate Casparian strip in original image and corresponding magnification of the endodermal region (insets). Asterisks indicate passage cells. Plants grew for 14 (A,E) or 10 (B,C,D) days. co, parenchymatic cortex; en, endodermis; ep, epidermis; ex, exodermis; pc, pericycle; ph, phloem; xy, xylem. Scale bars: 100 µm.

In *A. thaliana*, endodermal suberization is assumed to be subsequent to lignification of the Casparian strip (Doblas et al., 2017). To evaluate whether our protocol could distinguish between these polymers, we combined it with BF-based lignin staining (Kurihara et al., 2015). In FY/BF co-stained roots, we were able to clearly identify the lignified Casparian strip and the suberin lamella as separate entities in the *L. japonicus* root endodermis (Fig. 2C). The emission signals of both dyes were visually separable (Figs 3 and 4), and we rarely observed colocalization of FY-stained suberin and BF-labelled lignin in root samples. Moreover, FY signal was absent from the xylem, whereas BF stained meta- and protoxylem (Fig. 4A). This implies specificity of the staining technique, confirms distinct accumulation patterns and is consistent with independent functions of suberin and lignin depositions (Barberon et al., 2016). Barrier deposition strategies in roots differ between plant lineages and species (Holbein et al., 2021). We thus extended the protocol to other species, aiming to cover a representative set of spermatophytic lineages (The Angiosperm Phylogeny Group, 2016) (Fig. S1; Fig. 3A-E). To enhance visualization of cell structures inside roots, we further implemented concurrent cellulose staining using Calcofluor White (CW) into the protocol. This allowed for simultaneous visualization of the primary cell wall of non-lignified, unsuberized cells, and, in line with the spectral properties of CW, CW staining did not interfere with suberin and lignin signals (Figs 3 and 4). For most of the tested species, triple staining with CW, FY and BF allowed a clear differentiation between primary and suberized, or lignified secondary cell walls such as the Casparian strip (Figs 3 and 4A-F). Standard single-photon confocal imaging techniques were sufficient to resolve root barrier features in most cases. However, to increase the depth of imaging in thick roots, we employed a multiphoton setup in *Solanum lycopersicum* (Fig. 3C) and *Brachypodium distachyon* (Fig. 3D). For a better understanding of the root cell wall composition, and to evaluate imaging limitations associated with whole-mount analyses, we performed triple CW, BF and FY staining on root cross-sections of the same species (Fig. 4A-E). Apart from xylem cell walls within the stele, *L. japonicus* (Figs 2C, 3A and 4A) and *A. thaliana* (Figs 3B and 4B) roots both possessed lignin depositions mainly in form of classical endodermal Casparian strips, and displayed endodermal suberin lamellae. The crop tomato (*S. lycopersicum* ecotype Moneymaker) showed no suberin deposition in the endodermis and only hardly detectable Casparian strips (Figs 3C and 4C). The establishment of a suberized exodermis was only sparsely observed in both *L. japonicus* and *S. lycopersicum* (Figs 3A,C and 4A,C). Whereas *L. japonicus* seems to have no continuous exodermis at all (Figs 3A and 4A), *S. lycopersicum* showed only rare and weak suberization but, in line with previous reports (Li et al., 2018), lignification of the exodermis (Fig. 4C). In the monocot *B. distachyon*, structures comparable to Casparian strips were rarely observed and only evident near the meristematic zone. This suggests that Casparian strips might be weakly pronounced or absent in these species, or that its chemical constituents are distinct, and not stainable by BF. In older developmental regions of *B. distachyon* roots, lignification encompassed entire endodermal cells (Figs 3D and 4D; Fig. S2A-D), indicating that lignin or lignin-based barriers may be serving distinct roles in this species. Strikingly, in cross-sections of *B. distachyon* roots (Fig. 4D), endodermal lignin depositions were mainly found on inner periclinal cell walls, whereas suberin predominantly lined outer periclinal cell walls, suggesting a polarity of lignin and suberin depositions. As in other species, the *B. distachyon* endodermis contained unsuberized passage cells (Fig. S2B,C), and, interestingly, cells lacking lignification were also observed (Fig. 3D; Fig. S2C). A further remarkable feature of *B. distachyon* roots was the existence of an exodermis with lignin and weak suberin depositions following a pattern reminiscent of Casparian strips (Fig. 4D; Fig. S2A). Among the species examined here, roots of the gymnosperm tree *Picea glauca* showed the highest degree of both suberin and lignin deposition (Figs 3E and 4E), with most cortical cell walls of 14-day-old treelets, including those of the endodermis, lignified and suberized. Notably, *P. glauca* showed high variability in cell wall composition depending on the developmental stage (Fig. S3A,B).

In summary, different plant species displayed distinct barrier patterns, ranging from defined endodermal Casparian strips with or without accompanying suberin lamellae, to near universal lignification and suberization of the root cortex. The presented protocol allows qualitative imaging of diverse root barriers, including both inner (endodermis with Casparian strip) and peripheral barrier types such as exodermal (Figs 3D,E and 4D,E; Figs S2A,D and S3B) and peridermal suberization (Figs 2B; Figs S4 and S5). However, reliable quantitative visualization of suberin seems not to be feasible using FY owing to fast bleaching of this dye under laser exposure. Further limiting quantitative evaluation of both lignin and suberin depositions in inner root tissues of whole mounts is the clearing efficiency, as in whole-mount tissue, incomplete clearing will result in compromised signal intensity. In cross-sections, we did not observe differences in staining efficiency between previously cleared and fresh tissue (Fig. S6A,B). We also observed that CW-based staining of primary cell walls in deeper tissue layers was of limited efficiency in whole-mount samples, but uncompromised when root sections were stained. Using properly cleared samples, a 3D reconstruction of endodermal cell wall modification can be achieved using this protocol (Fig. S7). To identify optimal settings for specific staining signals, we determined the multiphoton excitation and emission spectra of FY and BF (Fig. S8A,B).

How these different strategies respond to environmental triggers and what their effects are on biotic interactions in the rhizosphere are exciting questions. We tested our protocol on a broad phylogenetic range of species inhabiting diverse ecological niches, including non-models and cultivated crops. In *L. japonicus*, nodulation symbiosis triggers the *de novo* formation of bacteria-filled nodule organs. This process is accompanied by the establishment of novel barrier types, such as a suberized periderm surrounding the entire nodule organ (Fig. 2B; Fig. S5). It will be interesting to examine further these symbiosis-related barrier structures and their biological roles. Allowing for co-observation of multiple cell wall components and fluorescent proteins in the same samples, the presented toolset represents a valuable advance towards addressing these questions, and provides an exciting handle for comparative exploration of the interplay between polymeric root barriers and rhizospheric composition and interactions.

## MATERIALS AND METHODS

### Plant and bacterial resources

Plants used in this study were *Lotus japonicus* ecotype Gifu B-129 (Handberg and Stougaard, 1992), *Arabidopsis thaliana* ecotype Col-0, *Solanum lycopersicum* ecotype Moneymaker (Dörffling, 1970), *Brachypodium distachyon* ecotype BD-21 (Garvin et al., 2008) and *Picea glauca* (1a Saatgut; http://www.1a-saatgut.de/). For analysis of symbiotic roots, *L. japonicus* plants were inoculated with *M. loti* MAFF303099 expressing *DsRED* (Maekawa et al., 2009).

### Plant growth and infection

All plants used in this study, except those shown in Fig. S4, were grown under sterile culture conditions. *A. thaliana* seeds were sterilized by 30 min incubation in a solution of 70% ethanol and 0.05% Triton X-100. Seeds of other species used in this study were sterilized by incubation in sodium hypochlorite solution containing 10 g/l Cl, then washed six times and incubated on a shaker in sterile ddH_2_O at room temperature until imbibed. Seeds were transferred to sterile growth media on square plastic dishes and stratified at 4°C in darkness. Following cold treatment, seeds were pre-germinated at 22°C in darkness, or directly transferred to growing conditions at 21°C in the light/17°C in the dark (16 h light, 8 h dark). Detailed growth conditions of individual species are listed in Table S1. For infection of *L. japonicus* with *M. loti*, liquid bacterial cultures were grown for 2 days at 28°C and pelleted for 10 min at 3000 ***g***. The bacterial pellet was washed twice and resuspended in quarter-strength B&D (Broughton and Dilworth, 1971) medium. For inoculations, the optical density at λ=600 was adjusted to 0.01 and 20 µl bacterial suspension were applied to each root. Roots were harvested after 10-21 days depending on the species (Table S1) for fixation, cuts or direct staining.

### Semi-thin sections of roots and nodules

Sectioning was conducted on either fresh, or previously fixed and cleared, primary root or nodule tissue. For sectioning, roots were cut into pieces of about 1 cm length, which contained the region of interest. These root fragments were embedded in 5-7% agarose. After hardening, small blocks of agarose including the sample fragments were sectioned by hand using a fresh razorblade.

### Confocal microscopy

Roots were analysed with Leica SP8 inverted (Figs 1 and 2A,B), Zeiss LSM 880 (Figs 3A,B,E and 4; Figs S2-S7) or Leica SP8 FALCON-DIVE (Figs 2C and 3C,D) confocal microscopes. 2D and 3D reconstructions were created using Leica LAS X or ZEN Blue software. For single-photon microscopy, settings for visualization of dyes were: objectives 10×/0.3 dry or 20×/0.8 dry; excitation (EX) 405 nm, emission (EM) 425-475 nm for CW; EX 488 nm, EM 520-550 nm for FY; EX 561 nm, EM 600-700 nm for BF in sequential scans. For multi-photon microscopy, settings for visualization of dyes were: objective 25×/0.95, water immersion; MP set at 1045 nm, MP2 (output power 2.24 W) set at 977 nm; fluorescence collected at 500-535 nm for FY, and 585-605 nm for BF.

### Fixation procedure

For fixation, root tissue was immersed in 4% paraformaldehyde in 1× PBS and gently shaken overnight at 4°C. Alternatively, tissue was immersed in 4% paraformaldehyde in 1× PBS and vacuum infiltrated for 1 h. After fixation, samples were washed three times in 1× PBS. Fixed samples were directly used for clearing.

### Clearing of fixed samples

We cleared the samples using a ClearSee-based protocol (Kurihara et al., 2015). ClearSee solution was prepared by dissolving xylitol powder (10% w/v), sodium deoxycholate (15% w/v) and urea (25% w/v) in water, without heating the solution. Previously fixed tissue was incubated in ClearSee at room temperature for 1-14 days until clear. To improve the clearing, the tissue was gently shaken and the ClearSee solution was regularly exchanged when discoloured. Clearing duration was highly dependent on plant species, age and tissue (for details, see Table S1).

### Staining procedure

For staining using dye combinations, best results were obtained when dyes were applied in the order BF, FY and CW. All dyes were successfully used either directly on thin cuts of fresh tissue, or on whole roots following clearing. For lignin staining, tissue was immersed in 0.2% BF in ClearSee for at least 1 h, rinsed once in fresh ClearSee and incubated in a second rinse for 30 min or longer. For suberin staining, a working solution of 0.01% FY in ethanol was prepared using a stock of 1% FY in DMSO. Tissue was rinsed once in ddH_2_O and immersed in FY working solution for 30 min at room temperature. For basic staining of cell walls, tissue was immersed in 0.1% aqueous solution of CW and incubated for 15 min. If thin cuts were used as starting material, incubation times for all staining and washing steps were reduced to 10 min.

If only FY staining was applied, roots were optionally counterstained using 0.5% (w/v) Aniline Blue in ddH_2_O for 20 min. Counterstaining of FY-stained samples with Aniline Blue improves contrast in thin samples and cuts, but is not recommended for imaging of deep tissue such as *L. japonicus* nodules to achieve higher signal intensity in optical sections.

Following the final staining, the tissue was washed once in 50% ethanol, twice in ddH_2_O and stored in 50% glycerol. When stored cool and dark, samples could be imaged for up to 3 weeks without significant signal loss. Note that counterstaining with Aniline Blue is not recommended when FY staining is combined with BF and/or CW. FY solutions and FY-stained tissue must be kept in darkness to prevent bleaching.

For a short guide to the triple-staining procedure, see supplementary Materials and Methods.

### Determination of dye spectra

Excitation and emission spectra for BF and FY were determined separately using a Leica SP8 FALCON-DIVE confocal microscope. *L. japonicus* Gifu nodules (21 days post-infection) were fixed, cleared, stained and sectioned as described. For both dyes, multi-photon excitation spectra were determined between λ=800 nm and 1265 nm, with stepwise increase of 15 nm; emission λ=500-550 nm for FY, λ=600-650 nm for BF. Emission was measured from λ=380 nm to 750 nm, at a 10 nm step size. For excitation, previously determined excitation maxima (λ=935 nm for FY, 1055 nm for BF) were used. For each of two independent replicates using sections of different nodules, five regions of interest at the nodule vascular endodermis were selected to quantify BF or FY signals.

## Supplementary Material

Supplementary information

Reviewer comments
