# Supplementary material for: Visualizing polymeric components that define distinct root barriers across plant lineages
Source: Development. 2021 Dec 8;148(23):dev199820. doi: 10.1242/dev.199820 (PMC8714062; doi:10.1242/dev.199820)
Supplement: Supplementary information [file develop-148-199820-s1.pdf]

Supplementary Materials and Methods

Short Guide: Triple staining of cell wall components

This protocol is optimized for fixed and cleared tissue (steps 1-4). Thin samples and tissue cuts can be stained directly without prior fixation or clearing. In this case start at step 5 of the protocol.

The procedure is optimized for triple staining of root tissue using Fluorol Yellow (FY), Basic Fuchsin (BF) and Calcofluor White (CW). If not all three dyes are needed, the respective steps can be skipped. Additional ClearSee-compatible dyes can potentially be implemented.

The procedure is compatible with imaging of fluorescet proteins and fluorescently labelled microorganisms.

For more details, see Methods section of the associated manuscript.

Buffers and solutions

A. 1x PBS: Dissolve NaCl [137 mM], KCl [2.7 mM], Na_2HPO_4 [10 mM] and KH_2PO_4 [1.8 mM] in ddH_2O. Adjust pH to 7,4 using 1 M HCl.
Note: Can be prepared as 10x stock.

B. Fixation buffer: Dissolve 4 % (w/v) PFA in 1x PBS, adjust pH to 6.9. Store at -20°C.

C. ClearSee solution: Dissolve xylitol powder [10% (w/v)], sodium deoxycholate [15% (w/v)] and urea [25% (w/v)] in ddH_2O. Do not heat. Store at room temperature.
Note: It can take several hours until components are fully dissolved. Best prepare the solution in a fume hood using a capped bottle.

D. FY stock solution: 1% FY in DMSO. Store at 4°C in darkness.
Note: Careful heating to 60°C may be required to aid dissolution.

E. FY working solution: Use the FY stock solution (D) to prepare a 0.01% FY solution in EtOH (96%). Store at 4°C in darkness.

F. BF working solution: Dissolve 0,2% Basic Fuchsin in ClearSee. Store in darkness.

G. CW working solution: Dissolve 0.1% CW in ddH_2O. Alternatively, commercially available staining solutions can be used directly. Store in darkness.

Development • Supplementary information

Staining procedure

1. Fix samples by 1h of vacuum infiltration in fixation buffer.
   Alternatively, incubate samples overnight at 4°C in fixation buffer.
2. Discard the fixation buffer and wash 3 times in 1x PBS.
3. Transfer fixed tissue to ClearSee solution, incubate by gently shaking at room temperature.
4. Exchange ClearSee solution regularly, until solution remains clear.
   Note: The Clearing process is highly dependent on the sample properties, and can take several days to weeks.
5. Lignin staining: Immerse tissue in BF working solution for at least 1h.
   Discard solution rinse twice in ClearSee. Keep in the second wash for at least 30 minutes. Tissue can be stored in ClearSee at this stage.
6. Suberin staining: Briefly rinse tissue once in ddH2O, then immerse in FY working solution and incubate 30 minutes at room temperature, discard FY solution.
   Optional: Incubate tissue in 0,5% Aniline Blue in ddH_2O for 20 min, discard solution.
   Note: Aniline Blue stains cell walls and can be used in combination with FY, to enhance contrast. It is not recommended to combine it with CW and BF staining, or for staining of deep tissue layers.
7. Cell wall staining: Immerse tissue in CW working solution and incubate for 15 minutes at room temperature, discard solution.
8. Quickly wash once in 50% EtOH and twice in ddH_2O.
9. Store at 4°C in darkness. For optimal signal intensity, image within three weeks.
   Mount tissue on slides using 50%-70 % glycerol to prevent drying.
   Note: To avoid bleaching, store FY-stained tissue in darkness whenever possible. Imaging of cuts should be done using low laser intensity, or fast scanning modes.

Development • Supplementary information

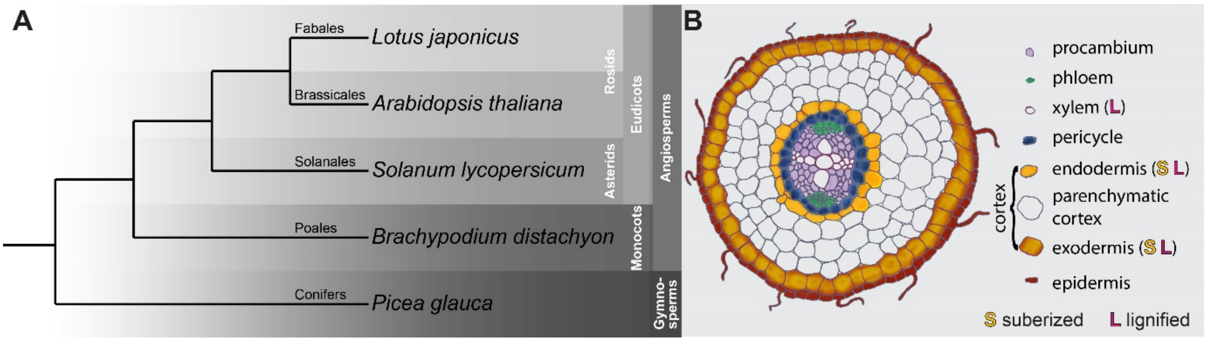

Fig. S1. Phylogenetic context of tested species and cell types visible in representative mature root section. (A) Phylogenetic relationships are based on the Angiosperm Phylogeny Group (APGIV, 2016). (B) Sketch of a root section showing commonly present cell types of a mature *A. thaliana* root. (B) shows a basic cell structure of a root during primary growth with two xylem poles and an established exodermis. The number of xylem poles varies between species and even within. Also not every species develops an exodermis. Common suberin and lignin depositions are indicated next to the cell types.

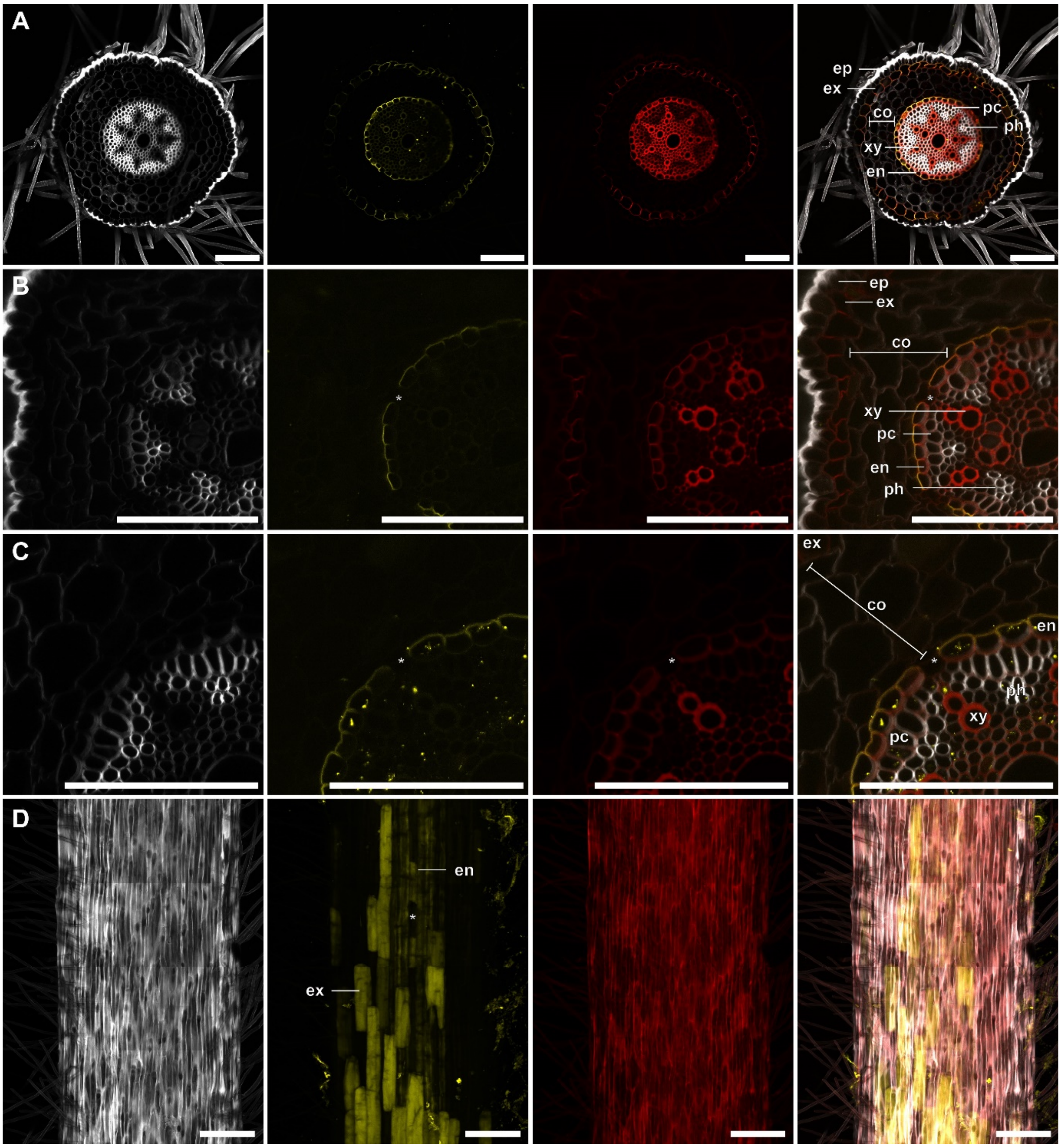

Fig. S2. Triple staining of cell wall components in *Brachypodium distachyon.* (A-D) Basic Fuchsin (BF), Fluorol Yellow (FY) and Calcofluor-white (CW) triple-stained semi-thin cross sections (A-C) and whole root mounts (D) of *B. distachyon* 10 days post germination. Panels (left to right) show CW, FY, BF and merged channels. (B-D) * indicate passage cells. Cell types indicated are co, parenchymatic cortex; en, endodermis; ep, epidermis; pc, pericycle; ph, phloem; xy, xylem and ex, exodermis. Scale bars: 100 µm.

Development • Supplementary information

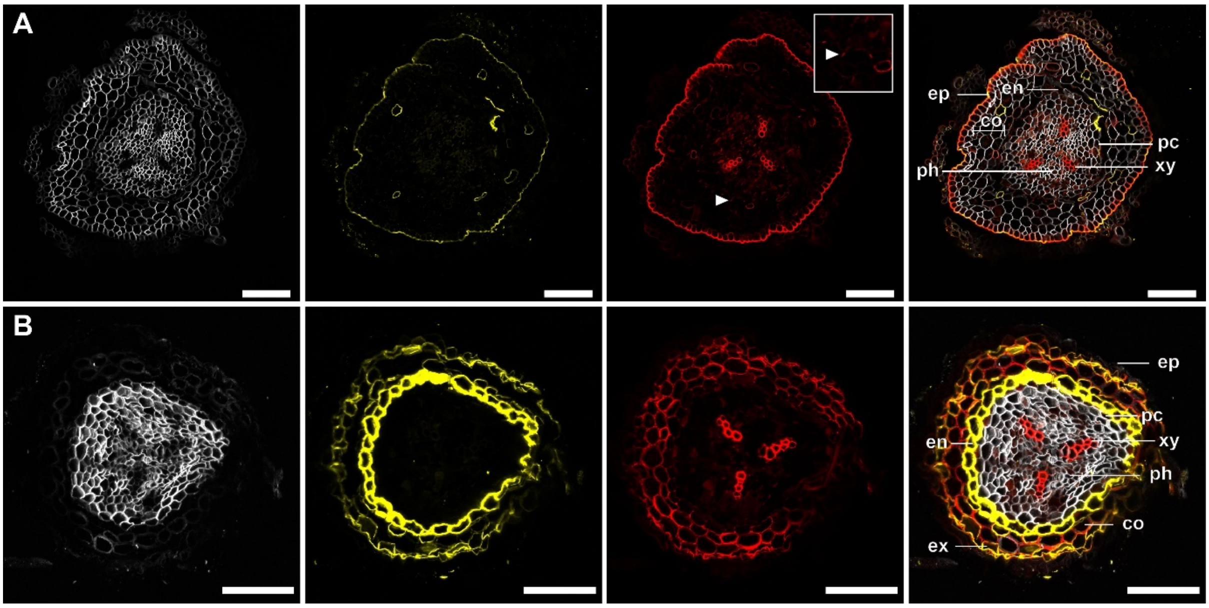

Fig. S3. Triple staining of cell wall components in *Picea glauca* cross sections (manual). (A-B) Basic Fuchsin (BF), Fluorol Yellow (FY) and Calcofluor-white (CW) triple-stained cross sections of *P. glauca* (A) 10 days and (B) 14 days post germination. Panels (left to right) show CW, FY, BF and merged channels. White arrowheads indicate Casparian strip in original image and corresponding magnification. Indicated cell types are co, parenchymatic cortex; en, endodermis; ep, epidermis; pc, pericycle; ph, phloem; xy, xylem; ex, exodermis. Scale bars: 100 µm.

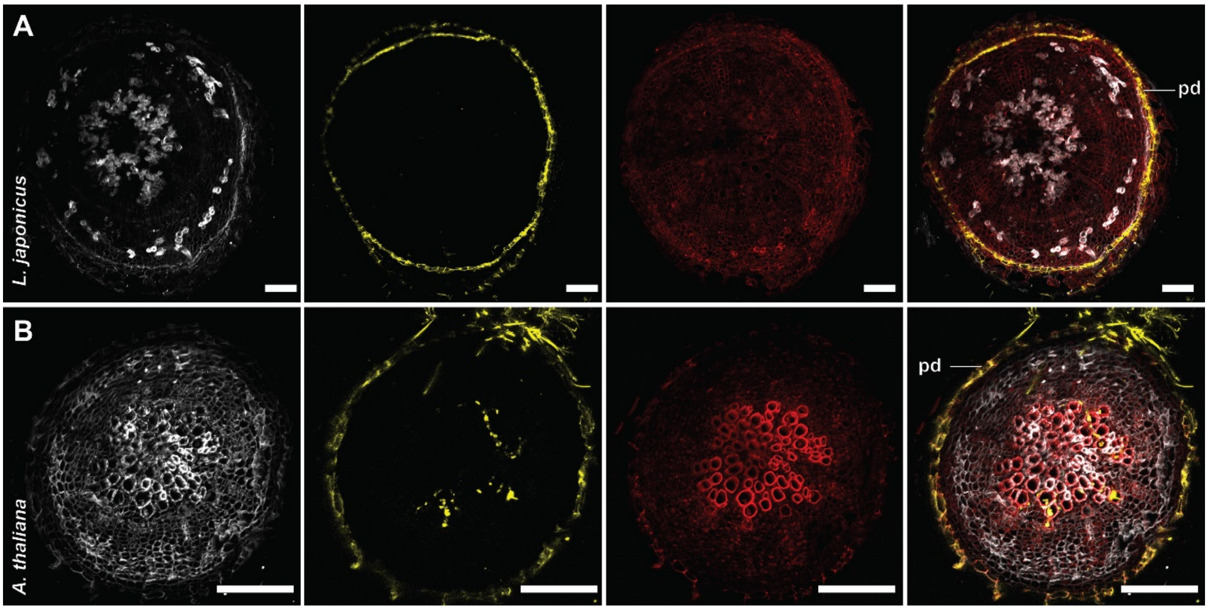

Fig. S4. Triple staining of cell wall components in semi-thin cross sections after secondary root growth. (A-B) Basic Fuchsin (BF), Fluorol Yellow (FY) and Calcofluor-white (CW) triple-stained cross sections of (A) *L. japonicus* and (B) *A. thaliana*. (A-B) cross sections of adult plants grown in pots. (A-B) Panels (left to right) show CW, FY, BF and merged channels. Suberized periderm (pd) stained by FY. Scale bars: 100 µm.

Development • Supplementary information

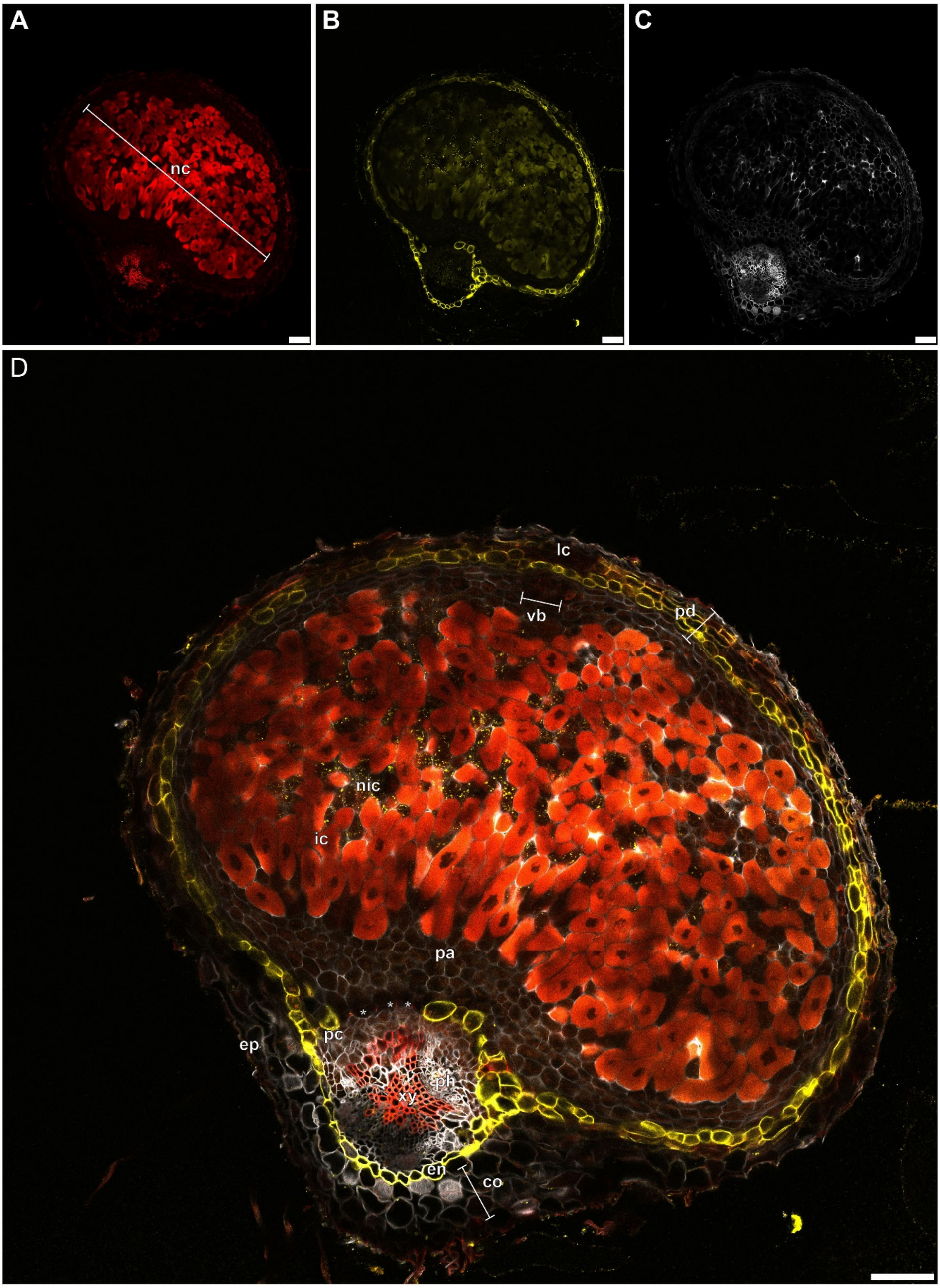

Fig. S5. Triple staining of nodule cross section. (A-B) Basic Fuchsin (BF), Fluorol Yellow (FY) and Calcofluor-white (CW) triple-stained cross section of a *L. japonicus* nodule (21 days post infection). (A) CW, (B) FY, (C) BF and *DsRED* expressed by *M. loti* and (D) merged channels. Cell types indicated are co, parenchymatic cortex; en, endodermis; ep, epidermis; pc, pericycle; ph, phloem; xy, xylem; pd, periderm; pa, nodule parenchyma; ic, infected cells; nic, non-infected cells; lc, lenticels; nc, nodule cortex and vb, vascular bundle. * indicate passage cells. Scale bar: 100 µm.

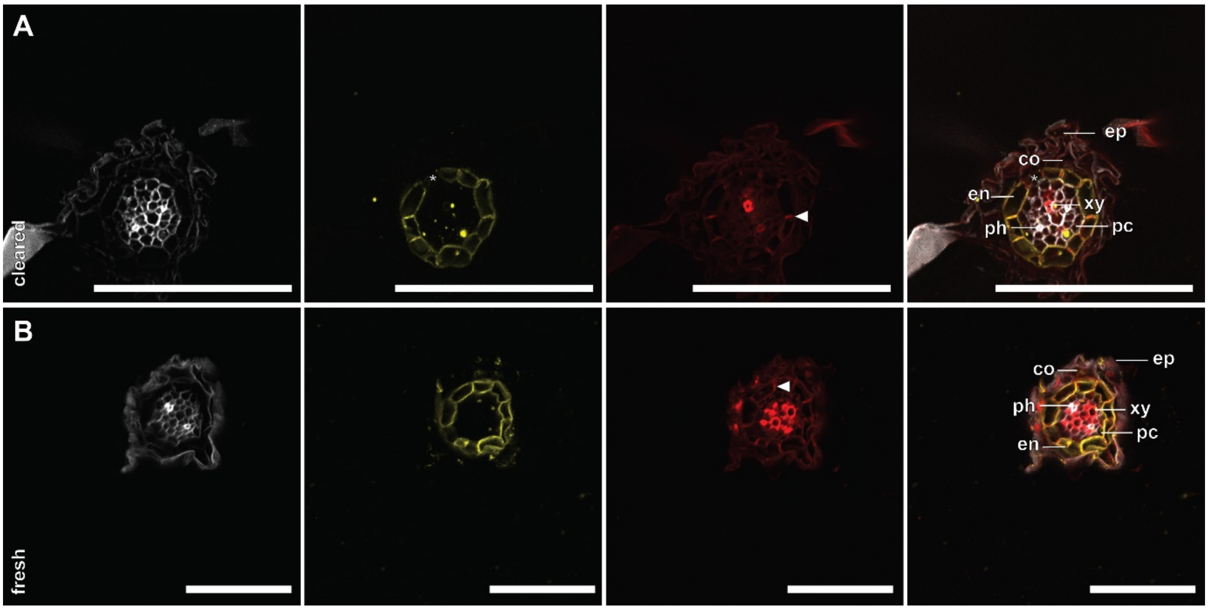

Fig. S6. ClearSee treatment does not compromise integrity and visualization of secondary cell wall components. (A-B) Basic Fuchsin (BF), Fluorol Yellow (FY) and Calcofluor-white (CW) triple-stained cross sections of (A) previously cleared and (B) fresh *A. thaliana* root cross sections (manual cuts), 10 days post germination. (A-B) Panels (left to right) show CW, FY, BF and merged channels. White arrowheads indicate Casparian strips, * indicate passage cells. Indicated cell types are co, parenchymatic cortex; en, endodermis; ep, epidermis; pc, pericycle; ph, phloem; xy, xylem. Scale bars: 50 µm.

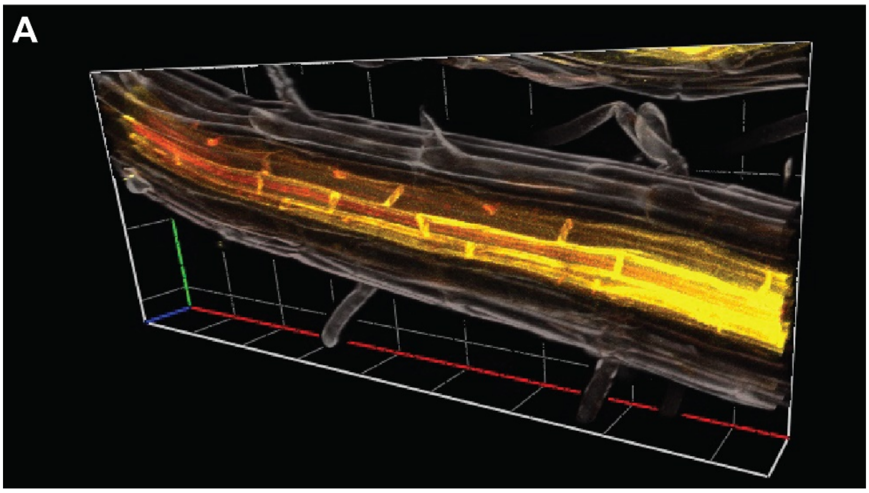

Fig. S7. 3D reconstruction of triple stained *A. thaliana* root. Computational 3D reconstruction of Basic Fuchsin, red; Fluorol Yellow, yellow; and Calcofluor-white, grey triple-stained *A. thaliana* root 10 days post germination. 3D-image reconstructed using Zen Blue 3.4 and 49 single images.

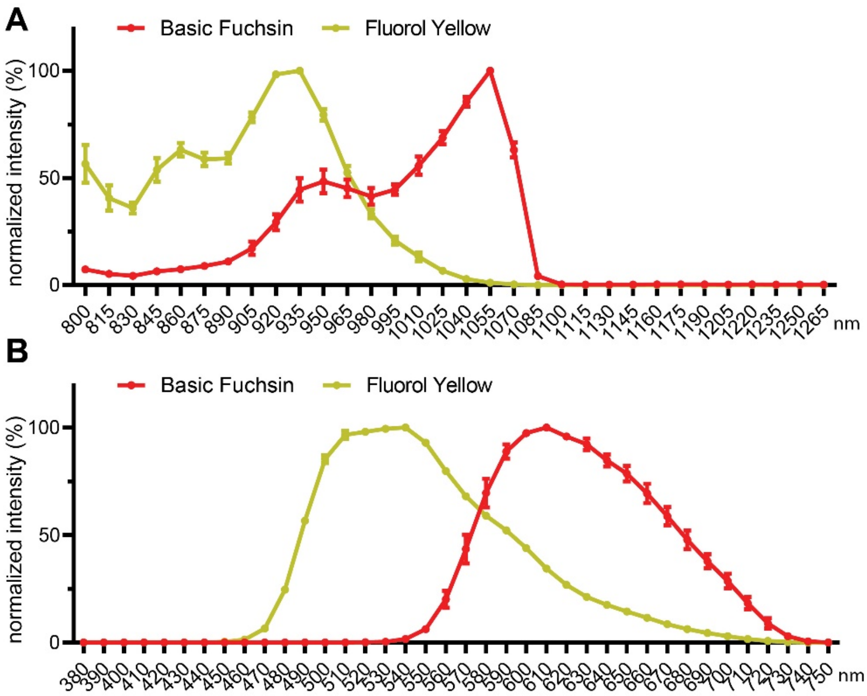

Fig. S8. Emission and excitation spectra of Fluorol Yellow (FY) and Basic Fuchsin (BF). (A) Multi photon excitation and (B) emission spectra of BF and FY in a stained *L. japonicus* nodule section. (A,B) The wavelength showing the highest intensity was set as 100%. Dots represent mean intensities at a given wavelength, error bars show standard deviation. n = 5 independent measurements on different regions near the nodule vasculature of the same nodule section. Similar results were acquired from two independent spectrum analyses, each measuring five spots, performed on two different nodules.

Development • Supplementary information

Table S1. Plant Cultivation Procedure. Cultivation procedure of different plant species used in this study.

**Plant species**	***Lotus japonicus***	***Arabidopsis thaliana***	***Solanum lycopersicum***	***Brachypodium dystachion***	***Picea glauca***
**Ecotype or source**	Gifu B-129	col-0	Money Maker	BD-21	1a seeds
**pre treatment**	scarification*	-	-	removal of husk and lemma**	-
**Sterilization Method and Duration**	Bleach treatment 10 min	Ethanol treatment 30 min	Bleach treatment 20 min	Bleach treatment 20 min	Bleach treatment 20 min
**Stratification at 4°C**	3 days	2 days	3 days	3 days	4 weeks
**pre germination*** **	Yes, 3 days	No	No	No	Yes, 1 week
**growth medium**	1/4 B&D****	1/2 MS	1/4 B&D****	1/4 B&D****	1/4 B&D****
**growth duration**	10-21 Days	10 Days	10 Days	10 Days	10-14 Days
**recommended clearing duration**	> 3days for roots; >1 week for nodules	> 1 day	>3 days	>1 week	>2 weeks

* seeds scratched with sandpaper prior to sterilization

** seeds soaked in water, lemma and husk removed using forceps prior to sterilization

*** germinated on separate plates for 3 days before growth period

**** (Broughton and Dilworth 1971).

Supplementary Reference

Broughton, W. J. and Dilworth, M. J. (1971). Control of leghaemoglobin synthesis in snake beans. *Biochem J* **125**, 1075-1080.
