# Supplementary material for: Visualizing polymeric components that define distinct root barriers across plant lineages
Source: Development. 2021 Dec 8;148(23):dev199820. doi: 10.1242/dev.199820 (PMC8714062; doi:10.1242/dev.199820)
Supplement: Reviewer comments [file develop-148-199820_review_history.pdf]

Visualizing polymeric components that define distinct root barriers across plant lineages

Moritz Sexauer, Defeng Shen, Maria Schön, Tonni Grube Andersen and Katharina Markmann
DOI: 10.1242/dev.199820

Editor: Yka Helariutta

Review timeline

Original submission:	20 May 2021
Editorial decision:	5 August 2021
First revision received:	6 September 2021
Accepted:	11 October 2021

Original submission

First decision letter

MS ID#: DEVELOP/2021/199820

MS TITLE: Visualizing polymeric components that define distinct root barriers across plant linages

AUTHORS: Moritz Sexauer, Defeng Shen, Maria Schön, Tonni Grube Andersen, and Katharina Markmann

I have now received all the referees' reports on the above manuscript, and have reached a decision. The referees' comments are appended below, or you can access them online: please go to BenchPress and click on the 'Manuscripts with Decisions' queue in the Author Area.

As you will see, the referees express considerable interest in your work, but have some significant criticisms and recommend a substantial revision of your manuscript before we can consider publication. If you are able to revise the manuscript along the lines suggested, which may involve further experiments, I will be happy receive a revised version of the manuscript. Your revised paper will be re-reviewed by one or more of the original referees, and acceptance of your manuscript will depend on your addressing satisfactorily the reviewers' major concerns. Please also note that Development will normally permit only one round of major revision.

We are aware that you may be experiencing disruption to the normal running of your lab that make experimental revisions challenging. If it would be helpful, we encourage you to contact us to discuss your revision in greater detail. Please send us a point-by-point response indicating where you are able to address concerns raised (either experimentally or by changes to the text) and where you will not be able to do so within the normal timeframe of a revision. We will then provide further guidance. Please also note that we are happy to extend revision timeframes as necessary.

Please attend to all of the reviewers' comments and ensure that you clearly highlight all changes made in the revised manuscript. Please avoid using 'Tracked changes' in Word files as these are lost in PDF conversion. I should be grateful if you would also provide a point-by-point response detailing how you have dealt with the points raised by the reviewers in the 'Response to Reviewers' box. If you do not agree with any of their criticisms or suggestions please explain clearly why this is so.

© 2021. Published by The Company of Biologists under the terms of the Creative Commons Attribution License (https://creativecommons.org/licenses/by/4.0/).

Reviewer 1

Advance summary and potential significance to field

This manuscript reports a new method to examine apoplastic barriers that are important for plant roots to function. This novel toolset combines improved tissue clearing, staining of multiple cell wall components while preserving fluorescent proteins, as well as single- and two-photon microscopy. The results are a striking improvement in visualizing the root anatomy of diverse species, both model and non-model. The advancement allows the authors to extend the proposed Casparian strip deposition mechanisms, of step-wise deposition of lignin and suberin, from the model species Arabidopsis to non-model species. The value of the method for examining biotic interactions of roots with nitrogen-fixing bacteria, DsRED expressing strain of the rhizobacterium Mesorhizobium loti, is demonstrated by the demonstration of a suberized layer around the nodules. This really opens up some interesting possibilities for future research.

Overall, the quality of the work is high, and the paper is well-written with reasonable conclusions.

Comments for the author

1. It is disappointing that the figures lack appropriate labels of the anatomy e.g. Figure 1B label epidermis, cortex, endodermis, xylem, phloem. Figure 1C, epidermis, cortex, stele. Figure 2A, stele, nodule primordium. Figure 2B label stele, nodule. Figure 2C epidermis, cortex, endodermis, xylem. Figure 3A label nodule, stele, cortex, epidermis (these could be done on the different channels were they are most visible, e.g. e for endodermis on the FY channel. Similarly in 3B and C, put an x for xylem on the BF channel, etc. In 3D label endodermis. I can't even tell what we are looking at in 3E. For Figure 4, labels could go on first colunm, just an e for endodermis in the FY channel, x for xylem in BF.

2. It would be nice to have some indication on the Figures themselves of what taxa is shown, e.g. an At Bd, Lj, etc on one corner of the merged image.

3. Figure legends should stand alone, so in each, expand FY, BF, CW on first use. In Figure 2B, why do the white arrowheads indicate infection threads, when the suberized periderm layer is the key finding here that should be indicated.

4. Figures 2B and 4F clearly show the suberized periderm around nodule, but it is not seen in 3A-is this a different developmental stage? Explain in the figure legend. Here, it appears there is lignin stained with BF around the nodule? Supplemental Figure S4 is the most beautiful illustration of the suberized nodule perhaps that should replace 3A?

5. In Figure 3C and D, it seems unusual that Brachypodium and Money Maker tomato were not stained with FY. In the cross-sections shown in Figure 4C and D, the specific pattern of FY label on the inner periclinal cell walls on Brachypodium seems inconsistent with the interpretation of the whole mounts,
"This suggests that these structures might be weakly pronounced or absent in these species, or that their chemical constituents are distinct, and not stainable by BF." Did the authors ever try to apply FY to fresh Brachypodium or tomato root cross sections? The ClearSee protocol could be extracting the suberin in the grass and tomato.

6. Methods of seed germination and plant growth are shown in Table S1, but it is never clearly stated if all of the roots were grown in sterile cultures, as Arabidopsis was.

Reviewer 2

Advance summary and potential significance to field

The manuscript "Visualizing polymeric components that define distinct root barriers across plant linages" by Moritz Sexauer and colleagues describes a method combining multiple stains to visualize cellulose, lignin and suberin in the same sample. Overall, this is a comprehensive

© 2021. Published by The Company of Biologists under the terms of the Creative Commons Attribution License (https://creativecommons.org/licenses/by/4.0/).

technique that promises to allow a simplified staining for multiple highly relevant polymers in roots in a variety of plant species. The manuscript is well written and composed.

Comments for the author

Development asks reviewers to consider two main questions. Firstly, what is the advance made in the paper and how significant is this for the field? Secondly do the data reported in the paper justify the conclusions drawn? Importantly, we ask that referees focus their suggestions for revision on those additions or changes necessary for potential acceptance of the manuscript, rather than on potential extensions of the study. Please refer to our Reviewer Guidelines for more details, and also consider the following points.

1. The main criterion for publication if a Research Article or Report in Development is that a paper should make a significant and novel contribution to our understanding of developmental mechanisms. Studies lacking such a contribution, no matter how meticulous, are not acceptable for publication.

2. Development has a 'Techniques and Resources' section. Articles submitted to this section should be assessed according to the novelty and importance for the community of the technique or resource reported.

3. Development is only able to accept around 30% of the papers it receives.
Editorial decisions are reached based on input from two or more usually three referees. Should the editor receive conflicting reports, he/she may contact you for further advice after your report has been submitted.

4. Development operates a 'cross-referee commenting' system, giving reviewers the option to view and comment on each others' reports before the editor makes a decision on the paper. Once all reports have been returned, you will receive an email inviting you to provide further feedback. We appreciate your participation in this process, which we find very helpful in making well-informed decisions and clearer guidance to authors.

5. We expect reviewers to review papers in a respectful manner and not to write anything that could cause offense or be defamatory. Please take care to ensure that any statements are factually supported, and that opinions stated are genuinely held and well-justified. On rare occasions where the editors of the journal are concerned that papers have not been reviewed according to these principles, we may contact the reviewer and request changes to the report before it is transmitted to the authors.
Thank you for contributing to the reviewing process and for your time and effort in helping to maintain Development as the most influential journal in its field.
Papers rejected from Development might be transferred, strictly with the authors' approval, to Biology Open, an online Open Access journal also published by The Company of Biologists. In this case, the reviewer reports and identities will be made available to the BiO Editors, who aim to make a decision on the basis of the existing reviews. Reviewer identities are always anonymous to authors. By passing on reports, our aim is to reduce the burden on authors and reviewers by avoiding the multiple rounds of review often encountered on a paper's route to publication. Please contact the Editorial Office should you have any queries.

Review
The manuscript “Visualizing polymeric components that define distinct root barriers across plant linages” by Moritz Sexauer and colleagues describes a method combining multiple stains to visualize cellulose, lignin and suberin in the same sample. Overall, this is a comprehensive technique that promises to allow a simplified staining for multiple highly relevant polymers in roots in a variety of plant species. The manuscript is well written and composed.
While this is really nice work, it is not entirely clear whether it is of sufficient novelty and importance for the community. Moreover, it is also unclear whether the protocol is efficient in staining non-endodermal suberin (e.g. periderm, exoderm, diffuse suberin).

© 2021. Published by The Company of Biologists under the terms of the Creative Commons Attribution License (https://creativecommons.org/licenses/by/4.0/).

Major:
1. Even though the authors have done exemplary work by looking into different species, it remains unclear whether all forms of suberin/tissues can be stained. What are the limitations of the stains? Can all tissues/forms of suberin be stained (e.g. exodermis, periderm, diffuse suberin)? Is the staining protocol qualitative or perhaps even semi-quantitative, which could be tested using different mutants in Arabidopsis like esb1 or gpat5?

Minor:
2. Abstract: "… but are efficient only in thin roots" That statement is unclear as cross-sections can also be efficiently stained in thicker roots.
3. Given the emphasis on suberin, the periderm should be covered in the introduction.
4. Page 3: "… Moreover, while A. thaliana indeed is a valuable model for image analysis,…" this seems to be an understatement.
5. Page numbers and line numbers would be helpful for reviewing the paper.
6. The authors make it seem that the discovery of suberin-rich tissue layers in nodules is novel. However, this has been reported previously, e.g.
Hartmann et al., 2020. Such work should be mentioned and references should be cited.
7. The figure legends should be improved. E.g. are the images based on an optical section from a confocal microscope? Consider to spell out abbreviations in figure legends to help reader.
8. The whole root mounts seem to be limited with regards to the depth of CW staining. Can one do a 3D reconstruction from z-stacks at least for some of the dyes? This and other limitations should be discussed.

First revision

Author response to reviewers' comments

Reviewer 1

Advance Summary and Potential Significance to Field:
This manuscript reports a new method to examine apoplastic barriers that are important for plant roots to function. This novel toolset combines improved tissue clearing, staining of multiple cell wall components while preserving fluorescent proteins, as well as single- and two-photon microscopy. The results are a striking improvement in visualizing the root anatomy of diverse species, both model and non-model. The advancement allows the authors to extend the proposed Casparian strip deposition mechanisms, of step-wise deposition of lignin and suberin, from the model species Arabidopsis to non-model species. The value of the method for examining biotic interactions of roots with nitrogen-fixing bacteria, DsRED expressing strain of the rhizobacterium Mesorhizobium loti, is demonstrated by the demonstration of a suberized layer around the nodules. This really opens up some interesting possibilities for future research.

Overall, the quality of the work is high, and the paper is well-written with reasonable conclusions.

Reviewer 1 Comments for the Author:
1. It is disappointing that the figures lack appropriate labels of the anatomy e.g. Figure 1B label epidermis, cortex, endodermis, xylem, phloem. Figure 1C, epidermis, cortex, stele. Figure 2A, stele, nodule primordium. Figure 2B label stele, nodule. Figure 2C epidermis, cortex, endodermis, xylem. Figure 3A, label nodule, stele, cortex, epidermis (these could be done on the different channels were they are most visible, e.g. e for endodermis on the FY channel. Similarly in 3B and C, put an x for xylem on the BF channel, etc. In 3D label endodermis. I can't even tell what we are looking at in 3E. For Figure 4, labels could go on first colunm, just an e for endodermis in the FY channel, x for xylem in BF.
Author response: Following the reviewers suggestion we have implemented annotations of anatomical features in Figures displaying both longitudinal mounts and cross sections. We thank for the suggestion, as the adjustments are an important improvement with respect to clarity.

© 2021. Published by The Company of Biologists under the terms of the Creative Commons Attribution License (https://creativecommons.org/licenses/by/4.0/).

2. It would be nice to have some indication on the Figures themselves of what taxa is shown, e.g. an At, Bd, Lj, etc on one corner of the merged image.
Author response: We included annotations in all Figures that feature more than one species.

3. Figure legends should stand alone, so in each, expand FY, BF, CW on first use.
Author response: We now define all abbreviations used in Figure legends at first use as suggested.

In Figure 2B, why do the white arrowheads indicate infection threads, when the suberized periderm layer is the key finding here, that should be indicated.
Author response: Figure 2B now features annotations of the suberized nodule periderm as well as other visible structures. An important aspect demonstrated here is the simultaneous visualization of fluorescent proteins - also in fine structures such as infection threads (ITs) - and suberin depositions. We therefore also retained labels pointing out ITs.

4. Figures 2B and 4F clearly show the suberized periderm around nodule, but it is not seen in 3A-is this a different developmental stage? Explain in the figure legend. Here, it appears there is lignin stained with BF around the nodule?
Author response: Nodules shown in Figures 2A and 3A are at a primordial stage, prior to periderm development. To clarify this, we indicated the stages of nodules shown in Figure 2A and 3A (primordium, 10 days post inoculation) as well as Figure 2B (mature nodule with periderm), and added a brief explanatory note with respect to the periderm to the Figure legends.

Supplemental Figure S4 is the most beautiful illustration of the suberized nodule, perhaps that should replace 3A?
Author response: As Figure S4 is a cross section, it does not match with the setup of Figure 3 (depicting longitudinal mounts). A nodule cross section featuring similar insights as Figure S4, albeit at smaller size, was shown in Figure 4F of the manuscript, but was removed to fit the journals figure formatting guidelines. Figure S5 now shows a nodule cross section at high resolution with appropriate annotations included.

5. In Figure 3C and D, it seems unusual that Brachypodium and Money Maker tomato were not stained with FY.
Author response: Under the growth conditions we used, the degree of suberization in *Brachypodium distachyon* and tomato ecotype Money Maker roots is weak (*B. distachyon*) to almost non-detectable (tomato Money Maker). In *B. distachyon*, suberization degree depends on the developmental stage and is mainly detected in mature parts of the root near the hypocotyl. In tomato, there are indications that suberization of apoplastic barriers is stress induced (Talano et al., 2006; doi.org/10.1016/j.jplph.2005.06.009), which could explain the near absence of suberin in young, tissue culture-grown plants. Similar observations have been made in grasses (Kreszies et al., 2918; doi.org/10.1111/nph.15351).

In the cross-sections shown in Figure 4C and D, the specific pattern of FY label on the inner periclinal cell walls on Brachypodium seems inconsistent with the interpretation of the whole mounts, “This suggests that these structures might be weakly pronounced or absent in these species, or that their chemical constituents are distinct, and not stainable by BF”.
Author response: The statement cited above refers to the Casparian strip, not to endodermal cell walls as a whole. To avoid misinterpretation, we have adjusted the wording as follows: ‘This suggests that Casparian strips might be weakly pronounced or absent in these species, or that its chemical constituents are distinct, and not stainable by BF’. (pages 5-6, lines 151-153, revised manuscript).

Did the authors ever try to apply FY to fresh Brachypodium or tomato root cross sections? The ClearSee protocol could be extracting the suberin in the grass and tomato.
Author response: We performed direct staining of untreated sections for several species, and did not observe obvious differences in suberin visualization compared to cleared root sections. This is consistent with the hydrophobic nature of suberin, which renders leakage into the aqueous ClearSee solution unlikely. While ClearSee treatment is not required for good staining results of cross sections, it is essential when analyzing whole mounts.

© 2021. Published by The Company of Biologists under the terms of the Creative Commons Attribution License (https://creativecommons.org/licenses/by/4.0/).

To demonstrate that suberin and lignin are retained during ClearSee treatment, we have included a direct comparison of previously cleared and directly stained, uncleared Arabidopsis sections in the revised manuscript (Fig. S6).

6. Methods of seed germination and plant growth are shown in Table S1, but it is never clearly stated if all of the roots were grown in sterile cultures, as Arabidopsis was.
Author response: We have added a statement to the Methods section of the manuscript clarifying that 'All plants used in this study except the ones shown in Fig. S4 were grown under sterile culture conditions.' (page 7, lines 200-201, revised manuscript).

Reviewer 2
The manuscript "Visualizing polymeric components that define distinct root barriers across plant linages" by Moritz Sexauer and colleagues describes a method combining multiple stains to visualize cellulose, lignin and suberin in the same sample. Overall, this is a comprehensive technique that promises to allow a simplified staining for multiple highly relevant polymers in roots in a variety of plant species. The manuscript is well written and composed.
While this is really nice work, it is not entirely clear whether it is of sufficient novelty and importance for the community. Moreover, it is also unclear whether the protocol is efficient in staining non-endodermal suberin (e.g. periderm, exoderm, diffuse suberin).

Major:
1. Even though the authors have done exemplary work by looking into different species, it remains unclear whether all forms of suberin/tissues can be stained. What are the limitations of the stains? Can all tissues/forms of suberin be stained (e.g. exodermis, periderm, diffuse suberin)? Is the staining protocol qualitative or perhaps even semi-quantitative, which could be tested using different mutants in Arabidopsis like esb1 or gpat5?
Author response: The protocol can efficiently stain a range of suberin containing tissues in different species, including exodermis and periderm. Exodermal suberin is featured in Fig. S2A,D (*Brachypodium distachyon*) and Fig. S3B (*Picea glauca*), and peridermal suberization is abundantly present and stained in *L. japonicus* nodules (Fig. 2B, Fig. S5). In the revised manuscript, we have further included an additional Figure to demonstrate staining of peridermal suberin in sections of pot-grown, mature roots of *Lotus japonicus* and *Arabidopsis thaliana* (Fig S4).
Quantification of staining results is limited mainly due to fast bleaching properties of Fluorol Yellow. We have included a paragraph in the Results and Discussion section where we discuss in detail the potentials and limitations of the described protocol and the dyes involved (page 6, lines 168-180, revised manuscript), and provide supporting Figure references (Fig S4; S6; S7). We further determined the excitation and emission spectra of basic fuchsin and fluorol yellow to confirm signal specificity (page 6 lines 179-180, revised manuscript; Figure S8).

Minor:
2. Abstract: "... but are efficient only in thin roots" That statement is unclear as cross-sections can also be efficiently stained in thicker roots.
Author response: To clarify, we have adjusted the text as follows: 'Techniques to label the respective polymers are emerging, but are efficient only in thin roots or sections.' (page 2, lines 24-25, revised manuscript).

3. Given the emphasis on suberin, the periderm should be covered in the introduction.
Author response: We have included a short introduction to the periderm in our revised manuscript (page 3, lines 60-63, revised manuscript).

4. Page 3: "... Moreover, while A. thaliana indeed is a valuable model for image analysis,..." this seems to be an understatement.
Author response: We have replaced 'valuable' by 'outstanding' in the respective sentence (page 3, line 81, revised manuscript).

5. Page numbers and line numbers would be helpful for reviewing the paper.
Author response: We have implemented both page and line numbers in the revised manuscript version.

© 2021. Published by The Company of Biologists under the terms of the Creative Commons Attribution License (https://creativecommons.org/licenses/by/4.0/).

6. The authors make it seem that the discovery of suberin-rich tissue layers in nodules is novel. However, this has been reported previously, e.g. Hartmann et al., 2020. Such work should be mentioned and references should be cited.
Author response: A reference to the respective publication is included (page 4, line 116, revised manuscript).

7. The figure legends should be improved. E.g. are the images based on an optical section from a confocal microscope? Consider to spell out abbreviations in figure legends to help reader.
Author response: We have added additional information to all Figure legends, including information on the developmental stages of the depicted plants. All abbreviations are now defined at first use in each legend. Please also see our response to comments # 3 and 4 by reviewer 1.

8. The whole root mounts seem to be limited with regards to the depth of CW staining. Can one do a 3D reconstruction from z-stacks at least for some of the dyes? This and other limitations should be discussed.
Author response: Yes, 3D imaging of whole mounts stained with the protocol described in this manuscript is possible. To exemplify this, we have included 3D images of Calcofluor-white, Fluorol Yellow and Basic Fuchsin stained *Arabidopsis thaliana* longitudinal root mounts (Figure S7A of the revised manuscript). The respective images were taken using Zeiss LSM880, and Zen Blue software was used to generate 3D images.

Second decision letter

MS ID#: DEVELOP/2021/199820

MS TITLE: Visualizing polymeric components that define distinct root barriers across plant linages

AUTHORS: Moritz Sexauer, Defeng Shen, Maria Schön, Tonni Grube Andersen, and Katharina Markmann
ARTICLE TYPE: Techniques and Resources Report

I am happy to tell you that your manuscript has been accepted for publication in Development, pending our standard ethics checks.

Reviewer 1

Advance summary and potential significance to field

This protocol paper will be well-cited. It opens up the world of simultaneously viewing of fluorescently labeled proteins and stains, deep within tissues.

Comments for the author

The authors have responded to all of my concerns. I think that the inclusion of the new labels on the Figures greatly improves the readability and interpretation of the data. I recommend publication of this revised version.

Reviewer 2

Advance summary and potential significance to field

The manuscript "Visualizing polymeric components that define distinct root barriers across plant linages" by Moritz Sexauer and colleagues describes a method combining multiple stains to visualize cellulose, lignin and suberin in the same sample. Overall, this is a comprehensive

© 2021. Published by The Company of Biologists under the terms of the Creative Commons Attribution License (https://creativecommons.org/licenses/by/4.0/).

technique that promises to allow a simplified staining for multiple highly relevant polymers in roots in a variety of plant species. The manuscript is well written and composed.
All concerns that I had raised were addressed by the authors and I commend them on their excellent manuscript.

Comments for the author

All important previous concerns have been addressed, however I realized that I had been not clear enough in one of the issues (see below)
4.Page 3: "… Moreover, while A. thaliana indeed is a valuable model for image analysis,…" this seems to be an understatement.
Author response: We have replaced 'valuable' by 'outstanding' in the respective sentence (page 3, line 81, revised manuscript).
What I meant was to consider something along the lines: Moreover, while A. thaliana indeed is a valuable model for many root architectural and developmental processes, as well as facilitates imaging and image analysis...

© 2021. Published by The Company of Biologists under the terms of the Creative Commons Attribution License (https://creativecommons.org/licenses/by/4.0/).
